# Development and Evaluation of the Veterinary Nurse Burnout Prevention Survey (VNBPS)

**DOI:** 10.3390/vetsci13010056

**Published:** 2026-01-07

**Authors:** Angela J. Chapman, Pauleen C. Bennett, Vanessa I. Rohlf

**Affiliations:** School of Psychology and Public Health, La Trobe University, Bundoora, VIC 3086, Australia; v.rohlf@latrobe.edu.au

**Keywords:** workplace wellbeing assessment, survey development, staff survey, burnout tool evaluation, leadership support, burnout prevention

## Abstract

Burnout results from chronic workplace stress and is most effectively addressed through workplace interventions. Successful implementation of interventions may, however, be constrained by factors within the work environment. This study aimed to evaluate a new tool, the Veterinary Nurse Burnout Prevention Survey (VNBPS). The 35-item, cross-sectional mixed methods survey aimed to identify the presence of burnout risk factors for veterinary nurses (VNs), and support selection of tailored interventions within the clinic. The VNBPS was conducted within VN teams (*N* = 67) across six Australian veterinary clinics between August and September 2025. After delivery and analysis of the survey, a summary of results and tailored recommendations was provided to each clinic. A subsequent evaluation questionnaire measured perceived ease of participation, accuracy of findings, and practicality of recommendations. The majority of the 17 respondents who completed the evaluation questionnaire (71%, *n* = 10) found the survey very easy to complete, and the findings to be accurate (79%, *n* = 11), or very accurate (21%, *n* = 3). Recommended interventions were perceived to be practical (50%, *n* = 7) or very practical (29%, *n* = 4). Internal reliability of the VNBPS was good. Participants reported that the survey held additional value in initiating conversations about burnout. This confirms the VNBPS as a useful tool to assist veterinary workplaces in the prevention of VN burnout and provide practical support for leaders to improve the wellbeing and professional sustainability of VN teams.

## Simple Summary

Burnout is the result of chronic work stress. Veterinary nurses (VNs) are at high risk of burnout, which can compromise individual wellbeing and organizational outcomes. Burnout prevention is, therefore, vital. Research in veterinary settings has identified multiple burnout risk factors and prevention strategies, but these are likely to vary among clinics. Workplace climate may also influence implementation of burnout prevention strategies. In this study, a 35-item survey, the Veterinary Nurse Burnout Prevention Survey (VNBPS), was designed to detect the presence of VN burnout risk factors and gauge workplace climate to inform tailored burnout prevention recommendations. The tool was piloted by 67 participants within six veterinary clinics. After delivery and analysis of the survey, a summary of results and tailored recommendations was provided. A follow up questionnaire then evaluated participants’ perceived ease of participation, accuracy of findings, and practicality of recommendations. Most participants perceived the survey as easy to complete. Participants also reported that the survey summary and burnout prevention recommendations were accurate, useful, and realistic. Participants commented that completing the survey helped facilitate additional conversations about burnout within the team. Overall, this tool can help veterinary clinics prevent burnout and support the wellbeing and long-term success of VN teams.

## 1. Introduction

Burnout is considered to be one of the top three issues currently facing the veterinary industry [1]. This is leading to increased staff turnover and a shortage of experienced veterinary nurses (VNs), a term used here to also refer to veterinary technicians and veterinary technologists [2]. Often conceptualized as a state of physical or psychological exhaustion, the characteristics of burnout are, in fact, more complex and have impacts beyond the affected individual [3,4]. In addition to feelings of fatigue, individuals may experience social and emotional effects, including irritability, withdrawal, low morale, pessimism, feelings of worthlessness, and a general inability to cope [4]. Physical consequences have been found to include cardiac disease, gastrointestinal disorders, hypercholesterolemia, type-2 diabetes, respiratory issues, headaches, and musculoskeletal pain [5]. Beyond these impacts, in a profession where VNs have an increased risk of suicide compared with the general population [6], burnout has been found to mediate the relationship between work related stress and suicidal ideation [7]. Prevention and management of burnout is, therefore, critical to VN wellbeing.

Beyond the individual, burnout can also affect patients, businesses, and co-workers. Burnout has been linked with increased self-reported medical errors, which, in addition to impacting patient outcomes, is likely to further decrease feelings of professional satisfaction in the individual and exacerbate burnout [8]. High levels of work stress and poor work–life balance have been linked with increased turnover in VNs, which may impact other team members through increased workload due to staff shortages, and loss of experienced mentors [9,10]. High levels of turnover and sick leave also result in lost revenue, and increased business costs related to recruitment and training [11]. Consequently, workplace awareness of burnout and employee wellbeing plays an essential role in promoting high-quality patient care and ensuring business sustainability.

Existing burnout interventions are frequently person-oriented and rely on the individual developing ways to alleviate workplace stress [12]. Examples of alleviation interventions include working less, developing coping skills through cognitive restructuring, practicing relaxation techniques such as mindfulness, and undergoing counseling or therapy [12]. Whilst these are beneficial as secondary prevention techniques, many rely on workplace flexibility to support their implementation and regular practice.

Primary interventions, aimed at addressing workplace drivers of burnout to prevent its onset at a team level, are relatively rare [12]. Previous research has identified the key workplace drivers of burnout in VNs. High workloads, lack of skill utilization, negative team culture, poor communication, and poor leadership were among those identified [9,13]. In addition, lack of schedule flexibility, poor remuneration, lack of progression opportunities, dealing with clients expressing rude or abusive behaviors, and lack of appreciation by management were also found to be key drivers of VN burnout [9,13]. To address these drivers, a range of primary intervention strategies have been developed by a panel of veterinary leadership and wellbeing experts [14]. The presence of workplace drivers of burnout, however, may vary between clinics, making it difficult for managers to know which drivers to target, should they wish to introduce burnout prevention programs in their workplace. To assist with selecting suitable strategies to prevent burnout, tools that support the early detection of workplace burnout drivers are reported as being valuable [15]. Such tools do not currently exist for VNs.

Successful implementation of interventions also relies on an understanding of the workplace climate, and potential barriers to implementing change [14]. Workplace climate describes the perceived characteristics of the workplace that are driven by behaviors, attitudes, and expectations of others [16]. Within these characteristics, barriers to implementing VN burnout prevention strategies have been proposed to include leadership quality, team culture, staff willingness for change, existing levels of burnout, financial capacity to implement change, and resources to support change [14]. Considering different perspectives when evaluating workplace climate is essential to identify disparities in workplace experiences between leaders and team members. Perceptions of leadership quality have been found to differ between leaders and their subordinates, with leaders rating their own performance more positively [17]. Addressing barriers related to leadership quality can be challenging if leaders do not perceive issues with their own performance. In addition, perceived psychological safety within veterinary clinics has been found to be impacted by hierarchical role and gender, with VNs reporting lower psychological safety scores compared with veterinarians and managerial staff, and women reporting lower psychological safety than men [18]. Women comprise 94–97% of the VN workforce internationally [19,20,21]; thus, VN experiences of workplace safety are likely to be negatively impacted by both professional and demographic group differences.

A number of tools have been developed to evaluate the organizational climate within human healthcare settings [22,23]. However, none of these existing tools capture all factors identified as potential barriers to implementing burnout prevention strategies in VNs [14], and nor do they capture differences in perceptions between leaders and team members. This study aimed to develop and test a survey designed to identify the presence of burnout risk factors, as well as barriers to change within the organizational climate. This was achieved by first developing and piloting the survey, then providing tailored, clinic-specific, burnout prevention recommendations based on the survey findings, and finally, seeking feedback from participants around the ease of use and accuracy of the tool. Once validated, it is anticipated that this tool will support VN leaders to select and implement tailored burnout prevention strategies that are most likely to succeed within each unique organizational setting.

## 2. Materials and Methods

The Veterinary Nurse Burnout Prevention Survey (VNBPS) was developed and evaluated in four stages. 1. Development of the survey; 2. Piloting of the VNBPS within six veterinary clinics; 3. VNBPS data analysis from each clinic and development of tailored burnout prevention recommendations; 4. Evaluation of the VNBPS and recommendations through a follow up evaluation questionnaire. Ethics approval for the study was obtained from the Human Ethics Low Risk Committee at La Trobe University, Australia (approval number HEC25287). Results are reported following the Consensus-Based Checklist for Reporting of Survey Studies [24].

### 2.1. Stage 1: Development of the VNBPS

#### 2.1.1. VNBPS Overview

Based on existing research [9,13,14], the VNBPS aims to provide veterinary clinics with a simple tool to evaluate the presence of burnout risk factors for VNs, and identify any aspects of the workplace climate that may act as a barrier to preventing burnout. Analysis of the presence of burnout risk factors was determined by the ten key VN burnout risk factors identified by Chapman et al. [9,13]. In their mixed-methods study, the authors analysed responses from 172 VNs, across nine countries, to determine broad themes describing perceived burnout contributors to VNs to ensure wide coverage of specific issues in this population. These factors are broad terms, created specifically to incorporate workplace stressors related to a range of patient, co-worker, client, and organisational issues. Evaluation of workplace climate factors that may inhibit the success of burnout prevention strategies was determined by the six key workplace barriers identified by Chapman et al.’s [14] study, which explored workplace barriers to addressing burnout. Individual questions within each of the six workplace climate subscales were selected based on the themes and subthemes around workplace barriers that were identified within the research [14]. As one part of a comprehensive program of study which used a robust, mixed-method design to deeply understand the experiences of VNs [9,13,14], initial survey development was well informed by substantial existing evidence and, therefore, did not employ a mixed-methods approach requiring additional qualitative data.

#### 2.1.2. VNBPS Structure

The VNBPS uses a cross-sectional mixed-methods study design and consists of three sections. Two contain quantitative questions, with the third containing one free text question. The survey sections are outlined in Table 1. Sections 1 and 2 of the VNBPS contain 34 questions, selected from a range of existing validated workplace wellbeing instruments (see below). Each item is scored on a five-point Likert scale ranging from 1 (strongly disagree) to 5 (strongly agree). Questions from existing measures were adapted when necessary to fit this format. For example, “Does your work make enough demands on all your skills and abilities?” was reworded to “My work makes enough demands on all my skills and abilities”. In addition, items rated on a six-point Likert scale were changed to a five-point Likert scale for consistency. Section 3 includes one optional free text question providing participants with the opportunity to provide any further information they feel is important. Guidelines on how to respond to free text questions without providing identifying information are included within the survey, for example, not including names, specific workplace responsibilities, or writing in a way that could identify individuals.

The aim of the VNBPS is to measure the collective team perspective of the workplace climate, and any differences between team members and team leaders. Hence, two versions of the survey were developed, containing the same questions rephrased for either VN team members or VN leaders. An initial screening question asks participants whether they hold a team member or leadership role. Based on their response, they are directed to the relevant version of the survey. To ensure privacy, additional demographic information is not collected. Contact details for local mental health helplines are provided in the participant information and consent form, and at the end of the survey, in the event of any discomfort around disclosure of work experiences. The full VNBPS can be seen in Supplementary Materials S1.

#### 2.1.3. Item Selection

Questions were sourced from the following instruments. The source of each VNBPS question can be seen in Table 2. Wording was adapted, where necessary, to be relevant to the veterinary workplace.

Healthcare-OH Survey (H-OH) [22]: Thirteen items were selected from this measure, which is designed to evaluate organizational health in acute sector healthcare organizations. Items were selected for the VNBPS ‘Burnout risk factors’ scale (Section 1, items 1 and 7) and for several of the subscales in Section 2; ‘Quality of leadership’ (items 11–13), ‘Existing team culture’ (items 17–18), ‘Financial Health of the clinic’ (items 27–30), and ‘Level of resources to support change’ (items 32–34). Items included statements such as “My daily workload is not achievable”.

Veterinary Job Demands and Resources Questionnaire (Vet-DRQ) [25]: Three items were selected from this questionnaire, which is based on the Job Demands-Resources model [26] and is designed to measure the impact of job demands, job resources, and personal resources, on the wellbeing of veterinary professionals. Items were selected for the VNBPS ‘Burnout risk factors’ scale (Section 1, items 2, 8, and 10) and included statements such as “My work offers me opportunities for continued learning”.

Culture of Care Barometer (CoCB) [23]: Six items were selected from this instrument, which was developed to assess the perceived workplace culture among healthcare workers, in order to identify opportunities for improvement in the quality and safety of care provided to patients. Items were included in the VNBPS ‘Burnout risk factors’ scale (Section 1, items 3–5) and the ‘Existing team culture’ (items 15–16) and ‘Levels of resources to support change’ (item 31) subscales in Section 2. Items included statements such as “A positive culture is visible where I work”.

Resistance to Change Scale (RTCS) [27]: Four items, one from each of the four subscales, were selected from this questionnaire, which is designed to measure individuals’ dispositional tendency to resist change. All items were included in the VNBPS ‘Staff willingness for change’ subscale (Section 2, items 19–22), and included statements such as “Changing plans seems like a real hassle to me”.

Oldenberg Burnout Inventory (OLBI) [28]: Four items were selected from this scale, which is designed to measure burnout within occupational settings. All items were included in the VNBPS ‘Existing level of burnout’ subscale (Section 2, items 23–26), and included statements such as “There are days when I feel tired before I arrive at work”.

Researcher designed questions: Three questions (items 6, 9, and 13) were designed by the researchers where suitable questions could not be identified among existing validated measures.

Following initial development, the VNBPS was reviewed by six VNs to establish face validity and pilot test the questions. Face validity is a subjective assessment to determine whether items are relevant, clear, and suitable for the intended audience [29]. Minor amendments were made to address areas of potential confusion identified by the group. The items shown in Table 2 are the final items, as used in the main part of the study.

**Table 2:** VNBPS items and sources from which each item was adapted.

Survey Item		Source
Section 1: Presence of burnout risk factors		
1.	My daily workload is not achievable.	HOHS—Staff wellbeing subscale [22]
2.	My work makes enough demands on all my skills and abilities.	Vet-DRQ—Skills discretion subscale [25]
3.	A positive culture is visible where I work.	COCB—Organizational values subscale [23]
4.	I am kept well informed about what is going on in our team.	COCB—Team support subscale [23]
5.	I feel part of a well-managed team.	COCB—Team support subscale [23]
6.	There are often times when I am expected to put the clinic workload ahead of my work–life balance (e.g., not have a break, work overtime, or take shifts that don’t always fit in with my life outside of work).	Researcher designed
7.	People here are compensated adequately for the job they do (including wages and non-monetary rewards).	HOHS—Finance and investment subscale [22]
8.	My work offers me opportunities for continuing learning.	Vet-DRQ—Possibilities for professional development subscale [25]
9.	I regularly have to deal with clients who do not treat me with the appropriate respect and politeness.	Researcher designed
10.	I feel appreciated at work by my direct supervisor.	Vet-DRQ—Support from supervisor subscale [25]
Section 2: Evaluation of workplace climate		
Subscale 2a: Quality of leadership		
11.	I trust the managers to make the right decisions.	HOHS—Leadership and management subscale [22]
12.	General running of the clinic is haphazard and things work out by chance rather than proper planning.	HOHS—Efficiency subscale [22]
13.	I believe that people in leadership roles are adequately supported by their managers.	Researcher designed
14.	Communication between management and medical staff is poor.	HOHS—Communication subscale [22]
Subscale 2b: Existing team culture		
15.	When things get difficult, I can rely on my colleagues.	COCB—Relationships with colleagues subscale [23]
16.	Staff successes are celebrated by the clinic.	COCB—Organizational values subscale [23]
17.	There is friction or confrontation within my team.	HOHS—Strategy subscale [22]
18.	There is a ‘no-blame’ culture here.	HOHS—Patient safety subscale [22]
Subscale 2c: Staff willingness for change		
19.	I like to do the same old things rather than try new and different ones.	RTCS—Routine seeking subscale [27]
20.	If I were to be informed that there’s going to be a significant change regarding the way things are done at work, I would probably feel stressed.	RTCS—Emotional reaction subscale [27]
21.	Changing plans seems like a real hassle to me.	RTCS—Short-term thinking subscale [27]
22.	Once I’ve come to a conclusion, I’m not likely to change my mind.	RTCS—Cognitive rigidity subscale [27]
Subscale 2d: Existing level of staff burnout		
23.	There are days when I feel tired before I arrive at work.	OLBI (Exhaustion subscale) [28]
24.	After work, I tend to need more time than in the past in order to relax and feel better.	OLBI (Exhaustion subscale) [28]
25.	I can tolerate the pressure of my work very well.	OLBI (Exhaustion subscale) [28]
26.	I find my work to be a positive challenge.	OLBI (Disengagement subscale) [28]
Subscale 2e: Financial health of the clinic		
27.	People who manage the budget lack contact with clinical areas.	HOHS—Finance and investment subscale [22]
28.	There is enough money invested in patient care and clinical equipment.	HOHS—Finance and investment subscale [22]
29.	There is enough money invested in computer systems and technology.	HOHS—Finance and investment subscale [22]
30.	There is enough money invested in buildings and renovations.	HOHS—Finance and investment subscale [22]
Subscale 2f: Level of resources to support change		
31.	I feel able to ask for help when I need it.	COCB—Team support subscale [23]
32.	The management team are transparent, so it is easy to see what is being planned.	HOHS—Communication subscale [22]
33.	If there was a sudden unforeseen incident (e.g., fire or power cut), the clinic would cope very well.	HOHS—Resilience subscale [22]
34.	The clinic rarely puts new things in place to improve quality.	HOHS—Resilience subscale [22]

Abbreviations: HOHS = Healthcare-OH Survey; Vet-DRQ = Veterinary Jobs Demands and Resources questionnaire; COCB = Culture of Care Barometer; RTCS = Resistance to Change Scale; OLBI—Oldenberg Burnout Inventory.

### 2.2. Stage 2: Piloting the VNBPS

#### 2.2.1. Participants

Purposive sampling was used to recruit clinics through the researchers’ professional networks. Clinics were approached via email if they met the inclusion criteria, including being located within the State of Victoria, Australia, and employing a minimum of eight VNs to ensure anonymity in responses. Individual participants within the clinic were eligible to participate if they were employed as a VN, or VN manager/leader, at least 18 years of age, and able to read and write in English, the language of the survey. A rolling recruitment strategy was employed, and it was planned to conclude recruitment once data saturation for the follow up evaluation questionnaire was reached.

#### 2.2.2. Pilot Test Procedure

Prior to gaining participation consent from clinics, the primary author (AC) met with clinic managers to ensure a comprehensive understanding of the study. A link to the VNBPS, along with information about the study, was emailed to eligible individuals directly from the research team to mitigate concern regarding potential negative workplace impacts and increase engagement with the survey [30]. Participating clinics were also provided with posters including a QR code link to the VNBPS to promote participation. Participation in the study was voluntary and anonymous. Responses were collected using QuestionPro Research Edition (Austin, TX, USA) [31], over a period of two weeks for each clinic, between 27 August and 28 September 2025. Email reminders were sent by the research team one week, and again two days, prior to the survey closure. Reminders about the study were also sent by the clinic via internal communication platforms. Participants were required to confirm that they met the eligibility criteria prior to commencing the survey.

### 2.3. Stage 3: Analysis of VNBPS Data and Development of Recommendations

#### 2.3.1. Data Analysis

Leadership and team member scores for each section of the VNBPS were analyzed separately for each clinic, to allow for comparison between the two groups. Descriptive statistics were calculated, with means and standard deviations reported for each item. Data from open ended questions were analyzed using NVivo version 15 (Denver, CO, USA) [32], and coded using directed content analysis [33] to contextualize quantitative data. Responses with missing data were excluded from analysis. Mean scores for each item in Section 1, and each subsection in Section 2, were then ranked in order of highest to lowest perceived burnout risk, and workplace climate barrier, for both leaders and the VN team members. This enabled identification of key burnout risks and barriers in each clinic, as well as comparison of perspectives between leaders and VN team members.

#### 2.3.2. Development of Recommendations

Following analysis of the VNBPS data, a summary of findings was developed for each participating clinic. The summary included details of the top three burnout risk factors within that clinic, identified in Section 1 of the VNBPS, and the three most notable aspects of the workplace climate that may help, or hinder, efforts to effect change, identified in Section 2 of the VNBPS. Information on any divergence in VN and leadership teams’ perceptions, identified through comparison of mean scores, was also included. The extent of divergence was first considered, with variations greater than 1.0 between VN and leadership team within the same clinic indicating a different rating on the ‘Strongly disagree’ to ‘Strongly agree’ Likert scale. General divergence between VN and leadership teams with the same clinic was then evaluated to establish any patterns, for example, if a VN team held a more negative perception across all six aspects of the workplace climate compared with their leaders.

The researchers used these findings to guide selection and recommendation of burnout prevention strategies to address the top three burnout risk factors in each clinic. Recommendations were selected from the burnout preventions strategies developed by Chapman et al. [14], and were guided by the existence of barriers identified within the cinic. For example, where existing levels of burnout were identified as a barrier to implementing change, large changes requiring extensive effort from staff, such as introducing new technology to improve communication, were avoided. Instead, smaller scale solutions, such as introducing regular team and management meetings, were initially recommended to effect small wins and promote a gradual increase in the teams’ capcity to cope with change. Specific recommendations to address barriers and disparities in perceptions were not provided, as the aim of the survey was to support selection of strategies to prevent burnout in VNs. However, the findings around existing barriers and perceptions were reported in the results summary to raise awareness of any issues that were identified within the clinic. Initial meetings were held between the primary researcher (AC) and clinic managers to discuss the findings. This allowed for clarification of existing clinic policies and procedures to ensure that the recommendations were relevant. Where contextual information supported adaptation of the recommendations to increase suitability for the clinic, adjustments were made accordingly. For example, shelter clinic biosecurity protocols were confirmed prior to making recommendations that would require interaction, and potential disease transmission, between different teams.

### 2.4. Stage 4: Evaluation of the VNBPS and Tailored Clinic Recommendations

#### 2.4.1. Evaluation Questionnaire Structure

The evaluation questionnaire comprised nine items designed by the research team to evaluate the ease of VNBPS completion, perceived accuracy of the results, and how practical the recommendations were for implementation within the clinic. Two initial screening questions asked participants if they held a team member or leadership role, and whether they had completed the VNBPS. Participants were then directed to questions that asked them to rate aspects of the VNBPS, such as “How easy did you find the survey to complete?”, or a free text question “can you tell us why you did not complete the survey?”. Rating questions were scored on a Likert scale, ranging from 1 (very easy), to 5 (very difficult). Participants were then asked if they had read the summary of findings and were directed to questions that asked them to rate the summary such as “How accurately do you feel the summary reflects the workplace?”, “How practical do you believe that the recommendations are for your clinic?”, or a free text question “Can you tell us why you have not read the summary and recommendations?”. Rating questions were scored on a Likert scale, ranging from 1 (very accurately), to 5 (very inaccurately). Due to the cross-sectional nature of the survey, the implementation of recommendations was not evaluated. The full evaluation questionnaire can be seen in Supplementary Materials S2.

#### 2.4.2. Evaluation Procedure

The summary of findings was sent to all eligible participants in each clinic directly from the research team, with a link to the follow up evaluation questionnaire. A reminder email, to encourage completion of the evaluation questionnaire, was sent by the research team one week after the summary and recommendations were released, as well as by the clinic. Data collection ceased after 3 weeks.

#### 2.4.3. Evaluation Data Analysis

Evaluation questionnaire data for all participating clinics were combined, data was cleaned, and incomplete responses excluded. Descriptive statistics were calculated for each item, and conventional content analysis was used to code responses to open ended questions into categories [33]. Cronbach’s alpha was calculated using Jamovi version 2.5 (Sydney, Australia) [34], to determine the internal consistency of the burnout risk factor scale, as well as the six workplace climate subscales of the workplace climate scale.

## 3. Results

Six veterinary clinics were recruited, including general practice, shelter, multi-disciplinary referral, corporate, private, not-for profit, urban, rural, small animal, and mixed-practice clinics, to establish a diverse sample across a variety of clinical settings. VN team sizes ranged from 10 to 65 members, including VNs and VN leaders. Response rates for each clinic can be seen in Table 3 and ranged from 16% to 91% for the VNBPS, and 0% to 30% for the evaluation questionnaire. Reasons for non-response included lack of time (67%, *n* = 2) and not checking emails regularly (33%, *n* = 1).

The VNBPS took an average of 6 and a half minutes to complete, with response times ranging from 3 to 37 min. As expected, burnout drivers, and barriers to addressing burnout, varied between clinics, with seven of the ten burnout risk factors found to be in the top three concerns across the six clinics. Lack of schedule flexibility was the most common risk factor and was ranked in the top three for 83% (*n* = 5) of participating clinics. Other top risk factors included dealing with clients expressing rude and abusive behaviors (50%, *n* = 3), poor management 50% (*n* = 3), poor communication (33%, *n* = 2), lack of progression opportunities, (17%, *n* = 1), poor remuneration (33%, *n* = 2), and high workload (17%, *n* = 1). The existing level of burnout was a common barrier across 83% (*n* = 5) of clinics; however, all six clinics had moderate to high scores on willingness for change, indicating that staff resistance to change was unlikely to hinder efforts to prevent burnout in these clinics. Leadership performance was rated higher by VN leaders than VN team members in three (50%) of the participating clinics. In two out of these three clinics, leaders rated all six aspects of the workplace climate higher than VN team members, which may suggest that leaders who believe they are performing well, perceive the workplace climate in a more positive light. Conflicting participant responses within qualitative data from multidisciplinary clinics indicated that separate cultures or climates may exist between different teams. One participant reported that “Each team operates on their own and conflict can be seen with people’s perception of each team and how busy they are”, and another noted the different expectations between teams “One department is always told to chip in and help the other teams, but when that department is drowning it is not reciprocated”. This suggests that there may be value in administering separate surveys to specific VN teams within large multidisciplinary clinics.

**Figure 1:**
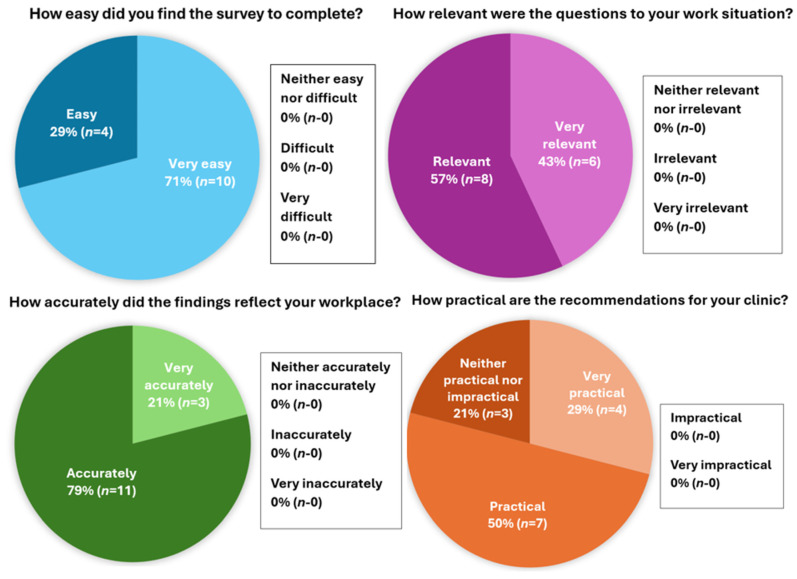
Evaluation questionnaire quantitative findings.

Usefulness of the tool was evaluated using data from the evaluation questionnaire, based on participants’ perceptions of the ease of completion, relevancy of the questions, accuracy of the findings, and practicality of the recommendations. Results can be seen in Figure 1. The VNBPS was considered to be easy to complete, with 100% of respondents rating it as either very easy (71%, *n* = 10), or easy (29%, *n* = 4). Twelve participants (71%) provided additional feedback in the optional free text question of the evaluation questionnaire. Themes can be seen in Table 4. Participants appreciated the accuracy of the findings and found them to be relevant to their workplace, with one participant reporting that “it was accurate and the recommendations were clear and informative” and another noting that the findings were “accurate, informative and interesting”. In addition to the basic functionality of the tool, qualitative responses in the evaluation questionnaire revealed the added benefit of opening a dialogue about burnout within the clinic. One participant noted that “It can be quite tricky to get an accurate gauge on the overall team. I feel like this has allowed feedback that may not otherwise have been voiced.”, and another observed that completing the VNBPS had been useful for “opening up a conversation within the hospital regarding burnout”.

The internal consistency of the VNBPS scales and subscales was good, with a mean Cronbach’s alpha coefficient of 0.73 for the ‘Burnout risk factor’ scale. Individual subscale mean values for Section 2 can be seen in Table 5 and ranged from 0.66 to 0.84. Two subscales, ‘Existing team culture’ and ‘Availability of resources’, fell below the acceptable recommendation of 0.7 for Cronbach’s alpha [35]. Measurement of Cronbach’s alpha on scales with less than 10 items is reported to be less reliable, with lower scores more commonly seen [35]. The subscales in the VNBPS contained only 4 items; therefore, inter-item correlations were also calculated. These evaluate the extent to which items are measuring the same phenomenon and provide an indication of item redundancy. Mean inter-item correlation values for these two subscales were 0.33 and 0.35, respectively, falling between the recommended 0.20 to 0.40 score [36]. This indicates that there is sufficient variance between the items to add value to the scale and warrant their retention.

## 4. Discussion

The purpose of this study was to develop and evaluate the usefulness of a tool to support veterinary clinics in selecting burnout prevention strategies with the greatest chance of success. The VNBPS was designed to identify the presence of burnout risk factors for VNs, as well as any workplace barriers that may negatively impact the ability to effect change.

The findings provide strong initial support for the use of the VNBPS. The majority of participants reported that the survey was easy to complete, provided an accurate assessment of the workplace, and that recommended interventions were realistic and practical. Positive responses were consistent among all participating clinics, suggesting that the VNBPS is equally effective across a range of clinical settings. Presence of burnout risk factors, and differences in perceptions of workplace climate between leaders and team members, were found across all clinics. This supports the need for a tool to assist with early detection of unique burnout drivers within individual clinics, and to provide leaders with insights into the experiences and perceptions of staff. Internal consistency of the VNBPS was good, indicating that the scales and subscales are reliable. Whilst some homogeneity was found between items on two of the subscales, there was sufficient variance between items to indicate that the questions still held value within the context of the scale.

In addition to the intended outcomes, the VNBPS was found to be beneficial in opening up a conversation about burnout within clinics. Lack of, or poor, communication has been found to be one of the top ten risk factors for burnout in VNs [13]. Honest conversation about burnout, with opportunities to be heard, has been proposed to be beneficial in helping to avoid burnout [37]. Furthermore, co-worker social support, when focused on seeking solutions rather than ruminating on the negative impacts of workplace stressors, has been found to buffer stress and burnout [38]. Use of the VNBPS may, therefore, have a secondary benefit of creating a positive dialogue within the team around proactively addressing stress and burnout.

Response rates for the VNBPS varied greatly between clinics. Smaller clinics, with VN teams of 13 members or less, showed greater response rates (62–91%), compared with larger clinics, with VN teams of 51 members or more across multiple teams, (16–35%). Survey response rates are generally declining [39], with reasons for non-response including survey length, lack of time, lack of interest in the topic, concern around the confidentiality of information, and a lack of belief that survey responses will lead to meaningful change [40,41]. Research suggests that inviting higher numbers of participants does not lead to greater response rates; however, identifying and targeting clearly defined populations may yield increased participation [42]. Furthermore, in large multidisciplinary clinics, idiocultures, a term used to describe distinctive subcultures within groups, are likely to exist at the team level due to local differences such as team structural characteristics, beliefs, and leader influence [43]. This was evident in the responses around the different management expectations of, and team member experiences within, teams from multidisciplinary clinics. In larger organizations, it is, therefore, recommended to survey each team individually, in order to examine workplace micro-climates and individual team issues that exist, rather than assuming the existence of a single ubiquitous culture.

In the current study, lack of time and not checking emails regularly were noted as reasons for non-response. Sending additional reminders using different communication methods, e.g., SMS, email, and workplace messaging platforms, may be of benefit in increasing response rates through leveraging individuals’ preferred communication style [44]. In addition, providing staff with time to complete the survey during their shift, as well as keeping the survey open long enough to allow for employees on leave to participate, can also help to increase response rates [45]. Response rates to research in human healthcare service providers are reported as variable [46] and is further reduced when focusing on mental health issues [47]. This is proposed to be related to fear of retribution or stigmatization [47]. Providing assurance of confidentiality and anonymity has been found to yield high response rates in research addressing the mental health of veterinary students, and so this should be a critical aspect for clinics utilizing the VNBPS. Increasing the perceived benefits of participation through providing incentives has also been found to be effective in increasing survey response rates [40]. It is important, however, that efforts to increase participation are not coercive, and are directed at passive non-responders, for example, individuals that have forgotten, did not receive the initial invitation, have been ill or absent, or have not yet had a chance to participate, rather than those who actively choose not to participate [48]. Forcing involuntary participation has ethical implications for individuals affected and can also affect the quality and authenticity of the data collected [49].

Primary interventions to address workplace drivers of burnout are rare [12], and VN leaders may lack the time, expertise, or support to fully understand the unique issues and requirements within their clinic. Implementing workplace change is challenging, with research suggesting that between 50% and 80% of organizational change initiatives fail [50]. Consequences of failed change initiatives extend beyond failure itself, and can lead to loss of trust in management, resentment towards the workplace, exhaustion, increased resistance to future change initiatives, increased turnover intentions, and negative impacts on workplace culture [51,52]. It is, therefore, important for leaders to have a thorough awareness of the burnout risk factors and barriers to change within their clinic, in order to maximize the success of interventions. This is of particular benefit in small clinics, where the use of the VNBPS, or other burnout surveillance measures, is challenging due to the lack of anonymity. Developing a culture of psychological safety is crucial in such clinics, to promote staff awareness of burnout and support voluntary disclosure of staff workplace difficulties. This study addresses a gap in the literature and presents a tool that can support VN leaders to identify the presence of burnout risk factors and select VN burnout interventions that have a greater chance of success. This reduces the burden on the individual to manage burnout through secondary, person-centred interventions aimed at managing workplace stress, and may lead to improvements in VN wellbeing, staff retention, and professional sustainability.

A key strength of this study was that the survey was based on information drawn from a comprehensive series of studies in the same occupation group. The ecological validity of the study is, therefore, high. The low response rates to the evaluation questionnaire, as well as to the VNBPS in some clinics, was a limitation. The purpose of this study was to undertake an initial evaluation, in a small number of clinics, to test the ease of participation and functionality of the VNBPS, and this was possible with the data collected. Based on these positive initial findings, further validation across a larger sample size, and in a wider geographical area, would be valuable to provide a more robust evaluation of the reliability of the VNBPS. In addition, this study lays the foundation for the development of similar tools to prevent burnout in other veterinary roles or professions at high risk of burnout. Whilst veterinarians and VNs may be exposed to similar environmental stressors, the control they have over those stressors differs due to their professional responsibilities. Translation of the survey to other professional roles, therefore, requires substantial preliminary research exploring the burnout risk factors and potential solutions specific to that role, to ensure that the tool addresses the unique job-related stressors. It should be acknowledged that veterinary, or other industry professionals, without any research background, may not have the skills or expertise required to conduct such work on their own. The research team for the current study benefited considerably from inclusion of mental health professionals skilled in survey development and research design.

## 5. Conclusions

Proactive identification and prevention of burnout is critical to increasing workplace wellbeing and retention of VNs within the veterinary profession. This study established the utility of an easy to administer tool to support early identification of burnout risk factors and selection of tailored strategies to address them within VN teams. In larger, or multidisciplinary, clinics, the VNBPS is likely to be most effective when administered to individual teams, as this will maximize response rates and account for separate cultures between teams. Participation may be increased through sending reminders via multiple communication methods and providing time for employees to participate while at work. However, participation must be voluntary and anonymous to avoid the risk of false or misleading data. Overall, the tool developed in this study can help VN leaders to apply previous research and make a practical difference to workplace systems and processes, as well as promote discussions and raise awareness of burnout within VN teams.

## Figures and Tables

**Table 1:** Items and purpose of sections within the VNBPS.

Survey Section	Purpose	Items
1	Measuring the presence of burnout risk factors	10 items, evaluating the presence of risk factors for burnout in VNs, identified by Chapman et al. [13]
2	Evaluating the workplace climate	24 items, comprising 6 subscales, each with 4-items evaluating the six aspects of the workplace climate, identified by Chapman et al. [14], that can impact the success of burnout prevention strategies. These include: (i) Leadership quality; (ii) Team culture; (iii) Staff willingness for change; (iv) Existing levels of burnout; (v) Financial capacity to implement change; (vi) Resources to support change.
3	Gathering additional context	One item, asking participants to provide any additional information about positive or negative aspects of the clinic culture that had not been covered in the previous sections.

**Table 3:** Participation for each clinic for the VNBPS and evaluation questionnaire.

Clinic	Total Clinic VN Leaders and Team Members	VNBPS	Evaluation Questionnaire
		Overall Response Rate *n* (Leaders, Team Members)	Overall Response Rate *n* (Leaders, Team Members)
1	65	23 (11, 12)	9 (5, 4)
2	11	10 (4, 6)	2 (0, 2)
3	13	8 (1, 7)	1 (0, 1)
4	13	10 (4, 6)	2 (1, 1)
5	10	8 (4, 4)	3 (1, 2)
6	51	8 (2, 6)	0 (0, 0)

**Table 4:** Themes arising from the evaluation questionnaire qualitative data.

Theme	Subthemes
The findings were positively received	Common descriptors included: Interesting Insightful Unsurprising
2. The summary was accurate	Relevant to staff concerns Accurately assessed the workplace
3. The recommendations were useful	Common descriptors included: Clear Helpful Useful
4. The process provided team members with a voice	Opened conversations Allowed feedback that would otherwise not be heard

**Table 5:** Cronbach’s alpha coefficients for each of the VNBPS scales and subscales.

VNBPS Scale	Cronbach’s Alpha
Section 1: Burnout risk factor	0.73
Section 2: Workplace Climate	
Subscale 2a: Quality of leadership	0.84
Subscale 2b: Existing team culture	0.66 *
Subscale 2c: Staff willingness for change	0.71
Subscale 2d: Existing level of staff burnout	0.72
Subscale 2e: Financial health of the clinic	0.81
Subscale 2f: Level of resources to support change	0.69 *

* Indicates subscales with Cronbach’s alpha below 0.7, which were followed up with inter-item correlations of 0.33 and 0.55, respectively.

## Data Availability

The datasets presented in this article are not readily available because of privacy restrictions. Requests to access the datasets should be directed to the corresponding authors.

## Supplementary Materials

The following supporting information can be downloaded at: https://www.mdpi.com/article/10.3390/vetsci13010056/s1, Supplementary Materials S1: Veterinary Nurse Burnout Prevention Survey (VNBPS); Supplementary Materials S2: Veterinary Nurse Burnout Prevention Survey—Evaluation Questionnaire.

## Abbreviations

The following abbreviations are used in this manuscript:

COCB	Culture of Care Barometer
HOHS	Healthcare-OH survey
OLBI	Oldenberg Burnout Inventory
RTCS	Resistance to Change Scale
Vet-DRQ	Veterinary Job Demands and Resources Questionnaire
VN	Veterinary Nurse (including Veterinary Technician and Veterinary Technologist)
VNBPS	Veterinary Nurse Burnout Prevention Survey
