# Supplementary material for: Development and Evaluation of the Veterinary Nurse Burnout Prevention Survey (VNBPS)
Source: Vet Sci. 2026 Jan 7;13(1):56. doi: 10.3390/vetsci13010056 (PMC12846452; doi:10.3390/vetsci13010056)
Supplement: Supplementary file 1 [file vetsci-13-00056-s001.zip › Supplementary materials S1.pdf]

Supplementary materials S1

Veterinary Nurse Burnout Prevention Survey (VNBPS)

Screening question

Are you a member of the veterinary nursing leadership team?

- ☐ Yes – *redirect to page 6 – veterinary nurse leadership survey*
- ☐ No – *redirect to page 2 – veterinary nurse team member survey*

Employee survey

Section S1

This section has 10 statements that will help us to determine the presence of risk factors for burnout in veterinary nurses within your clinic. Statements are rated on a 5-point Likert scale from 'Strongly disagree' to 'Strongly agree'.
Questions have been adapted from a range of existing validated wellbeing, and organisational evaluation instruments.

	Strongly Disagree	Disagree	Neither agree nor disagree	Agree	Strongly Agree
1. My daily workload is not achievable.					
2. My work makes enough demands on all my skills and abilities.					
3. A positive culture is visible where I work.					
4. I am kept well informed about what is going on in our team.					
5. I feel part of a well-managed team.					
6. There are often times when I am expected to put the clinic workload ahead of my work-life balance (e.g., not have a break, work overtime, or take shifts that don't always fit in with my life outside of work)					
7. People here are compensated adequately for the job they do (including wages and non-monetary rewards).					
8. My work offers me opportunities for continued learning.					
9. I regularly have to deal with clients who do not treat me with the appropriate respect and politeness.					
10. I feel appreciated at work by my direct supervisor.					

Section S2

This section has 24 questions that will help us to determine the presence of any barriers to addressing veterinary nurse burnout within your clinic. Questions are rated on a 5-point Likert scale from ‘Strongly disagree’ to ‘Strongly agree’.
In the following questions “managers” are people in your workplace that make organisational decisions that affect how you do your job. Depending on your clinic, managers may be different to your direct supervisors.
Questions have been adapted from a range of existing validated wellbeing, and organisational evaluation instruments.

	**Strongly Disagree**	**Disagree**	**Neither agree nor disagree**	**Agree**	**Strongly Agree**
11. I trust the managers to make the right decisions.					
12. General running of the clinic is haphazard and things work out by chance rather than proper planning.					
13. I believe that people in leadership roles are adequately supported by their managers.					
14. Communication between management and medical staff is poor.					
15. When things get difficult, I can rely on my colleagues.					
16. Staff successes are celebrated by the clinic.					
17. There is friction or confrontation within my team.					
18. There is a ‘no-blame’ culture here.					
19. I like to do the same old things rather than try new and different ones.					
20. If I were to be informed that there’s going to be a significant change regarding the way things are done at work, I would probably feel stressed.					
21. Changing plans seems like a real hassle to me.					
22. Once I’ve come to a conclusion, I’m not likely to change my mind.					
23. There are days when I feel tired before I arrive at work.					

24. After work, I tend to need more time than in the past in order to relax and feel better.					
25. I can tolerate the pressure of my work very well.					
26. I find my work to be a positive challenge.					
27. People who manage the budget lack contact with clinical areas.					
28. There is enough money invested in patient care and clinical equipment.					
29. There is enough money invested in computer systems and technology.					
30. There is enough money invested in buildings and renovations.					
31. I feel able to ask for help when I need it.					
32. The management team are transparent, so it is easy to see what is being planned.					
33. If there was a sudden unforeseen incident (e.g. fire or powercut), the clinic would cope very well.					
34. The clinic rarely puts new things in place to improve quality.					

Section S3

This final section gives you the opportunity to provide additional information about any positive or negative aspects of the culture in your clinic that have not been covered above. Workplace culture describes the values, behaviours, and attitudes of your managers and colleagues, which create the atmosphere in which you work. We will only share these comments in a format where all comments are grouped together in one file.

If you choose to write comments, we recommend that you take the following steps to protect your anonymity:

- Don't write in a way that identifies you
- Don't use your name or colleagues' names
- Don't say what area you work in or the work you are responsible for.

If there is any identifying information in the comments you write, we will remove or rephrase it in a way to protect your anonymity before sharing the collective comments.

Optional free text question: Would you like to tell us anything else about the positive or negative aspects of the culture or work systems in your clinic and how they impact your wellbeing? Please provide your answer in the box below.

Veterinary nurse leadership survey

Section S1

This section has 10 statements that will help us to determine the presence of risk factors for burnout in veterinary nurses within your clinic. Statements are rated on a 5-point Likert scale from 'Strongly disagree' to 'Strongly agree'.
Questions have been adapted from a range of existing validated wellbeing, and organisational evaluation instruments.

	Strongly Disagree	Disagree	Neither agree nor disagree	Agree	Strongly Agree
1. The daily workload of the veterinary nurse team is not achievable.					
2. The workplace makes enough demands on the skills and abilities of the veterinary nursing team.					
3. A positive culture is visible where I work.					
4. I keep the veterinary nurse team well informed about what is going on.					
5. The veterinary nurse team are well-managed.					
6. I believe there are often times when veterinary nurse team members should put the clinic workload ahead of their work-life balance (e.g., not have a break, work overtime, or take shifts that don't always fit in with their life outside of work)					
7. People here are compensated adequately for the job they do (including wages and non-monetary rewards).					
8. Veterinary nurse team members are offered opportunities for continued learning.					
9. Veterinary nurse team members regularly have to deal with clients who do not treat them with appropriate respect and politeness.					
10. I show my veterinary nurse team members that I value them.					

Section S2

This section has 24 questions that will help us to determine the presence of any barriers to addressing veterinary nurse burnout within your clinic. Questions are rated on a 5-point Likert scale from ‘Strongly disagree’ to ‘Strongly agree’.
In the following questions “managers” are people in your workplace that make organisational decisions that affect how VNTs do their job. Depending on your clinic, managers may be different to those in supervisory or leadership roles.
Questions have been adapted from a range of existing validated wellbeing, and organisational evaluation instruments.

	**Strongly Disagree**	**Disagree**	**Neither agree nor disagree**	**Agree**	**Strongly Agree**
11. The management team can be trusted to make the right decisions.					
12. General running of the clinic is haphazard and things work out by chance rather than proper planning.					
13. I believe that people in leadership roles are adequately supported by their managers.					
14. Communication between management and medical staff is poor.					
15. When things get difficult, the veterinary nursing team can rely on their colleagues.					
16. Staff successes are celebrated by the clinic.					
17. There is friction or confrontation within the veterinary nurse team.					
18. There is a ‘no-blame’ culture here.					
19. I like to do the same old things rather than try new and different ones.					
20. If I were faced with implementing a significant change regarding the way things are done at work, I would probably feel stressed.					
21. Changing plans seems like a real hassle to me.					
22. Once I’ve come to a conclusion, I’m not likely to change my mind.					
23. There are days when I feel tired before I arrive at work.					

24. After work, I tend to need more time than in the past in order to relax and feel better.					
25. I can tolerate the pressure of my work very well.					
26. I find my work to be a positive challenge.					
27. People who manage the budget lack contact with clinical areas.					
28. There is enough money invested in patient care and clinical equipment.					
29. There is enough money invested in computer systems and technology.					
30. There is enough money invested in buildings and renovations.					
31. The veterinary nurse team are able to ask for help when they need it.					
32. We are transparent with the veterinary nurse team, so it is easy for them to see what is being planned.					
33. If there was a sudden unforeseen incident e.g. fire or powercut, we would cope very well.					
34. We rarely put new things in place to improve quality.					

Section S3

This final section gives you the opportunity to provide additional information about any positive or negative aspects of the culture in your clinic that have not been covered above. Workplace culture describes the values, behaviours, and attitudes of your managers and colleagues, which create the atmosphere in which you work. We will only share these comments in a format where all comments are grouped together in one file.

If you choose to write comments, we recommend that you take the following steps to protect your anonymity:

- Don't write in a way that identifies you
- Don't use your name or colleagues' names
- Don't say what area you work in or the work you are responsible for.

If there is any identifying information in the comments you write, we will remove or rephrase it in a way to protect your anonymity before sharing the collective comments.

Optional free text question: Would you like to tell us anything else about the positive or negative aspects of the culture or work systems in your clinic and how they impact the wellbeing of yourself or the veterinary nurse team? Please provide your answer in the box below.

Scoring

Detailed scoring guidelines for the VNBPS can be found at www.veterinaryburnout.com.au
