# Supplementary material for: Development and Evaluation of the Veterinary Nurse Burnout Prevention Survey (VNBPS)
Source: Vet Sci. 2026 Jan 7;13(1):56. doi: 10.3390/vetsci13010056 (PMC12846452; doi:10.3390/vetsci13010056)
Supplement: Supplementary file 1 [file vetsci-13-00056-s001.zip › Supplementary materials S2.pdf]

Supplementary materials S2

Veterinary Nurse Burnout Prevention Survey – Evaluation Questionnaire

Earlier we contacted you about a study "*Development and evaluation of a Veterinary Nurse Workplace Burnout Prevention Survey*" that your clinic participated in. We'd like to now ask you some follow-up questions based on the summary of findings that you have received. These questions are designed to help us evaluate the effectiveness of the survey you completed.

Based on the summary of findings and recommendations that have been circulated to you, please answer the following questions.

1) Are you a member of the leadership team?
   i. Yes
   ii. No

2) Did you complete the survey
   i. Yes *(directed to question 3)*
   ii. No *(directed to question 5)*

3) How easy did you find the survey to complete?
   I. very easy
   II. easy
   III. neither easy nor difficult
   IV. difficult
   V. very difficult

4) How relevant were the questions to your work situation?
   i. very relevant
   ii. relevant
   iii. neither relevant nor irrelevant
   iv. irrelevant
   v. very irrelevant
   (directed to question 6)

5) Can you tell us why you did not complete the survey?

   [Free text box]

6) Have you read the summary of findings and recommendations?
   i. Yes *(directed to question 7)*
   ii. No *(directed to question 11)*

7) What did you think of the summary and recommendations?

[Free text box]

8) How accurately do you feel that the summary reflects the workplace

i. very accurately
ii. accurately
iii. neither accurately nor inaccurately
iv. inaccurately
v. very inaccurately

9) How accurately do you feel that the summary reflects the workplace culture?

I. very accurately
II. accurately
III. neither accurately nor inaccurately
IV. inaccurately
V. very inaccurately

10) How practical do you believe that the recommendations are for your clinic?

I. very practical
II. practical
III. neither practical nor impractical
IV. impractical
V. very impractical
(directed to exit the survey)

11) Can you tell us why you have not read the summary and recommendations?

[Free text box]
